# Investigation of the Combined Effect of Variable Inlet Guide Vane Drift, Fouling, and Inlet Air Cooling on Gas Turbine Performance

**DOI:** 10.3390/e21121186

**Published:** 2019-12-01

**Authors:** Muhammad Baqir Hashmi, Tamiru Alemu Lemma, Zainal Ambri Abdul Karim

**Affiliations:** Department of Mechanical Engineering, Universiti Teknologi PETRONAS, Bander Seri Iskandar, 31750 Tronoh, Perak Darul Ridzuan, Malaysia; baqirhashmi.123@gmail.com (M.B.H.); ambri@utp.edu.my (Z.A.A.K.)

**Keywords:** variable geometry, industrial gas turbine, variable inlet guide vane drift, fouling, inlet air cooling

## Abstract

Variable geometry gas turbines are susceptible to various malfunctions and performance deterioration phenomena, such as variable inlet guide vane (VIGV) drift, compressor fouling, and high inlet air temperatures. The present study investigates the combined effect of these performance deterioration phenomena on the health and overall performance of a three-shaft gas turbine engine (GE LM1600). For this purpose, a steady-state simulation model of the turbine was developed using a commercial software named GasTurb 12. In addition, the effect of an inlet air cooling (IAC) technique on the gas turbine performance was examined. The design point results were validated using literature results and data from the manufacturer’s catalog. The gas turbine exhibited significant deterioration in power output and thermal efficiency by 21.09% and 7.92%, respectively, due to the augmented high inlet air temperature and fouling. However, the integration of the inlet air cooling technique helped in improving the power output, thermal efficiency, and surge margin by 29.67%, 7.38%, 32.84%, respectively. Additionally, the specific fuel consumption (SFC) was reduced by 6.88%. The VIGV down-drift schedule has also resulted in improved power output, thermal efficiency, and the surge margin by 14.53%, 5.55%, and 32.08%, respectively, while the SFC decreased by 5.23%. The current model can assist in troubleshooting the root cause of performance degradation and surging in an engine faced with VIGV drift and fouling simultaneously. Moreover, the combined study also indicated the optimum schedule during VIGV drift and fouling for performance improvement via the IAC technique.

## 1. Introduction

Modern urbanization renders gas turbines to be a vital entity for power generation, aviation, and naval industries. Attributes including high efficiency, compact size, greater emissions compliance, wider fuel variability, flexibility in quicker ramp-up and shutdown, and cost-effectiveness help gas turbines to penetrate today’s market. On the other hand, the pursuit of higher reliability, availability, and sustainability demands modern gas turbines to incorporate advanced features [1]. Among such features is the variable geometry in compressor and turbines that includes variable inlet guide vane (VIGV), variable stator vanes (VSVs), variable bleed valve (VBV), and variable area nozzle (VAN) [2]. Variable guide vanes (VIGVs and VSVs) are made to function when the engine faces a speed fluctuation during startup, shutdown, and load change since they are scheduled as a function of spool speed [3]. Often at low speed, during startup and shutdown, the engine may experience surging and stalling that can be harmful to the engine’s overall health and performance [4]. Hence, the VIGVs and bleed control mechanism ensure stability and improve performance by alleviating surge in the axial compressor. In addition, the new generation of gas turbines face highly significant performance decline (deterioration) due to nonlinear and complex dynamics. Deterioration may also be triggered by some other factors such as malfunctions induced in the variable geometry mechanism, fouling in the axial compressor, and increased ambient temperature [5].

The VIGVs are controlled by an actuating mechanism via different linkages connected to guide vanes [6]. The VIVGs may encounter some malfunctions such as drifting of VIGVs away from the normal operational envelope. These malfunctions and faults lead to surging and choking in the compressor stages and eventually cause an accidental shutdown. Ishak and Ahmad [7] categorized the common phenomena responsible for VIGV drift as (i) high-speed stop, (ii) low-speed stop, (iii) hydraulic ram leakage, and (iv) Rotary variable displacement transducer (RVDT) misalignment. Similarly, Tsalavoutas et al. [8] stated that VIGVs are drifted outside from their normal schedule due to some other reasons: Wearing of actuation mechanism linkages and mispositioning/stacking of vanes due to loosed bolts. In order to avoid the occurrence of these faults, there is a need to continuously monitor and ensure that the movement and position of the actuation mechanism are synchronized with the actual design schedule. This approach; however, seems quite impractical as it is very hard to monitor the position of an actuation mechanism for a multiple numbers of vanes (in the case of vane stacking/loose bolt). Hence, Tsalavoutas et al. [8] developed an adaptive performance model, initially suggested by Stamatis et al. [9], to detect malfunctions in VIGVs. A few researchers have put forth efforts to observe the effect of VIGV drift on the performance of gas turbine by implanting some deliberate faults. Recently, Cruz-Manzo et al. [10] developed a MATLAB Simulink-based performance model to examine the effect of implanted VIGV offset position angle on the performance of the compressor. The VIGV faults were indicated by a comparison of the offset between the VIGV position demanded and position given by the control system during operation. Similarly, Ishak and Ahmad [7] developed a virtual sensor to predict the failure or malfunction of VIGVs. The fault was identified by comparing the deviation of the VIGV position given by the virtual sensor with the actual guide vane position from the control panel. Moreover, Razak et al. [11] discussed the importance of the condition monitoring system to detect the VIGV malfunctions. Although a few researchers have analyzed the effect of VIGV drift on the performance of gas turbine through implanting deliberate faults, there has not been a study that simulates the real VIGV drift that engine faces during operation.

Gas turbines also face performance deterioration due to compressor fouling and increased inlet air temperature. Compressor fouling happens when sticky particulate matters get deposited on the compressor’s annulus passage including rotors and stators [12]. Details on the effect of fouling on gas turbine performance can be found in [13,14,15]. Adaptive performance models and simulations were conducted to predict the fouling rate with the passage of operating time to avoid severe performance deterioration. Qingcai et al. [16] developed a genetic algorithm (GA)-based steady-state simulation model to analyse the effect of changing fouling parameters, mass flow, and isentropic efficiency on full- and part-load performance of a three-shaft industrial gas turbine. Similarly, Mohammadi and Montazteri-Gh [17] developed a fault simulation model by implanting some faults signatures through changing compressor and turbine characteristics curves. Although many researchers have worked on the fouling, no study was reported in the literature related to fouling severity for a variable geometry gas turbine. High inlet air temperature also leads to performance deterioration in the gas turbine. Normally, hot climates face a temperature augmentation of around 10 °C above the design condition temperature (i.e., 15 °C) that leads to a power deterioration by around 7% [18]. To avoid this power deterioration, inlet air cooling (IAC) is a commercially employed technique that reduces the temperature of the intake air to the compressor [19]. A variety of techniques were employed in the literature for IAC for industrial gas turbines, as discussed by Bakeem et al. [20]. Although a variety of pertinent literature exists in [21,22,23,24,25] regarding the IAC technique, to the authors’ knowledge, there is no evidence of any investigation of the effect of IAC on the performance and performance degradation characteristics of variable geometry industrial gas turbines. 

The VIGV drift and fouling generally depict similar behavior by reducing the annular flow passage of the compressor, consequently, causing the overall performance deterioration and compressor failure due to surging. Hence, it is a hard task to identify the root cause of the performance degradation and surging, which can be either VIGV drift or fouling. After a detailed study of the literature, it has become evident that the VIGV drift has remained barely studied. Although some researchers [8] have worked on it, their work focused on implanted VIGV drift faults. Besides, the effect of the real time VIGV drift that an engine faces during operation (i.e., incorporating the minimum and maximum limits of drift offset ranging from −6.5° to +6.5°, respectively) has not been simulated. Moreover, fouling and IAC have not been studied for a variable geometry gas turbine. The combined effect of VIGV drift, fouling, and IAC has also remained unexplored.

In the present study, the combined effect of VIGV drift, compressor fouling, and increased ambient temperature on gas turbine performance were investigated. A steady-state simulation model for a three-shaft gas turbine (GE LM1600) was developed using a commercial software, GasTurb 12. Firstly, the effect of VIGV drift was simulated by running the off-design steady-state model on three different variable geometry schedules: (i) up-drift schedule (off-set of +6.5° from Muir [26]), (ii) normal schedule (Muir schedule [26]), and (iii) down-drift schedule (off-set of −6.5° from Muir [26]). Secondly, a parametric study was conducted to simulate the effect of increased ambient temperature on the performance of the targeted engine. IAC was envisaged by a designated temperature (T = 265 K). Finally, the effect of compressor fouling on the performance of the gas turbine was simulated by using the modifier module available in the gas turbine. 

## 2. Thermodynamic Model

### 2.1. Model Inputs and Physical Properties 

The focus for the present paper was a three-shaft industrial gas turbine. Accordingly, we selected LM1600 engine since it involves variable geometry inlet guide vanes and its design is similar to other gas turbines like Rolls-Royce RB211 and MT30 engines. General Electric LM1600 engine is featured by ISO power rating of 13.7 MW with 50 Hz generator frequency, a low pressure (LP) spool, a concentric high pressure (HP) spool, and an aerodynamically coupled power turbine. The VIGV is installed at the low pressure compressor. The engine is typically utilized for power generation and mechanical drive applications in the oil and gas industry. The basic technical data needed for modeling the gas turbine are listed in Table 1.

In most cases, the thermodynamic processes involved in a gas turbine, such as compression, combustion, and expansion, are assumed to be ideal and the working fluid properties (specific heat *C*_p_ and isentropic index γ) are also considered constant. However, practically, these assumptions are not valid as the specific heat of the working fluid (i.e., air) varies with respect to temperature in the real process [27]. In addition, during combustion, air is converted into gaseous products having different composition that can affect the *C*_p_ and γ. Hence, GasTurb 12 considers the working fluid as a semi-ideal gas and the specific heats of air and the gaseous products to vary with temperature as given in Equations (1) and (2) [27,28].

(1) $$C_{p}=A+\frac{BT}{100}+C{\left(\frac{T}{100}\right)}^{-2},$$

(2) $$C_{pa}=0.7553\times C_{p,N2}+0.2314\times C_{p,O2}+0.0128\times C_{p,Ar}+0.0005\times C_{p,CO2},$$

where $C_{p,N2}$, $C_{p,O2}$, $C_{p,Ar},$ and $C_{p,CO2}$ are the specific heat values estimated from Equation (1) using the values of the coefficients *A*, *B*, and *C* from Table 2.

### 2.2. Design Point Calculations

**Figure 1:**
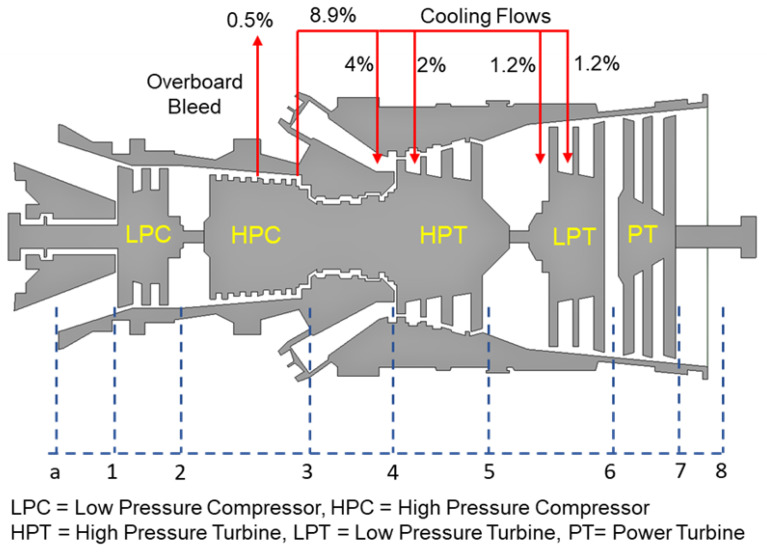
Schematic of the three-shaft gas turbine with cooling flow.

Design point calculation is a basic step during the performance evaluation of any gas turbine, because it helps in finding the unknown design parameters using thermodynamic and compatibility equations. This calculation requires the inherent design characteristics of a specific engine to be re-established. However, due to the scarcity of data and very limited information made public through literature or via product brochures, some empirical assumptions and engineering judgments were required, as given in Table 3. The power rating and spool speeds are in accordance with the information in the gas turbine product catalog and the ambient conditions data are based on the international organization for standardization (ISO) design point standards. The overall thermodynamic modeling of a gas turbine is often established through individual component-based modeling. GasTurb 12 also works on the same principle of component-based modeling. Generally, a three-shaft gas turbine is comprised of eight major components: Inlet duct, low-pressure compressor (LPC), high-pressure compressor (HPC), combustor, high-pressure turbine (HPT), low-pressure turbine (LPT), free power turbine (FPT), and an exhaust duct at the end. These components were; thus, modeled exclusively during design point by utilizing input parameters values that were provided by the manufacturer and some assumed values. In addition, GasTurb 12 also requires secondary air flows data: turbine cooling bleed and overboard bleed flow data [29]. In the present study; however, the HPT and LPT were cooled with bleed extracted from downstream of HPC, as shown in Figure 1.

The individual gas turbine components are generally modeled using physical- and thermodynamic-based equations. In addition, component characteristics maps are utilized to find the values of the mass flow, pressure ratio, and efficiency of the compressor and turbine. Once the inlet conditions (*m_a_, T_a_, P_a_*) and fuel flow are known, the unknown parameters can be found at every station using thermodynamic equations. The component-wise thermodynamic equations needed for modeling are discussed below.

For inlet duct, the pressure loss is accounted as follows:

(3) $$P_{1}=P_{0}\times \left(1-\;\Delta PR_{inlet}\right).$$

For the LP compressor, with known inlet conditions (*T*_1_, *P*_1_) and a given compressor map, the exit conditions (*T_2_, P_2_, Wc, m_2_*) can be easily estimated. The outlet temperature (*T*_2_) is calculated using the specific entropy correlation in Equation (4) to account for the effect of temperature on the specific heat of the working fluid.

(4) $$\Psi \left(T_{2s}\right)=\Psi \left(T_{1}\right)+\ln\left(\frac{P_{2}}{P_{1}}\right).$$

(5) $$T_{2s}=T_{1}+\left(\frac{T_{1}}{\eta_{lpc}}\right)\left(PR_{lpc}^{\frac{\gamma_{a}-1}{\gamma_{a}}}-1\right).$$

(6) $$T_{2}=T_{1}+\frac{T_{1}}{\eta_{c,isen\;}}\left(\left(\frac{T_{2s}}{T_{1}}\right)-1\right).$$

The compressor power consumption can be estimated by the following expressions:

(7) $${\dot{W}}_{lpc}=\;{\dot{m}}_{1}\left(h(T_{2}\right)-h(T_{1})),$$

where the values of *η*_lpc_ and *PR*_lpc_ can be determined from the specific compressor map at a certain shaft speed *N*. Similarly, for HPC, the modeling equations are as follows:

(8) $$\Psi \left(T_{3s}\right)=\Psi \left(T_{2}\right)+\ln\left(\frac{P_{3}}{P_{2}}\right).$$

(9) $$T_{3s}=T_{2}+\left(\frac{T_{2}}{\eta_{hpc}}\right)\left(PR_{hpc}^{\frac{\gamma_{a}-1}{\gamma_{a}}}-1\right).$$

(10) $$T_{2}=T_{1}+\frac{T_{1}}{\eta_{c,isen\;}}\left(\left(\frac{T_{2s}}{T_{1}}\right)-1\right).$$

(11) $${\dot{W}}_{hpc}=\;{\dot{m}}_{2}\left(h(T_{3}\right)-h(T_{2})).$$

Th combustion chamber is modeled using the simplified energy balance equation and pressure loss expression. Assuming the value of combustion efficiency, the temperature *T*_4_ can be estimated as follows:

(12) $$\eta_{cc}=\frac{\left({\dot{m}}_{3}+{\dot{m}}_{f}\right)C_{pg}\left(T_{4}\right)-{\dot{m}}_{3}C_{pa}\left(T_{3}\right)}{{\dot{m}}_{f}\times LHV}.$$

(13) $$P_{4}=P_{3}\times \left(1-\;\Delta PR_{cc}\right).$$

The HPT can also be modeled in the same fashion as the compressor:

(14) $$\Psi \left(T_{5s}\right)=\Psi \left(T_{4}\right)+\ln\left(\frac{P_{4}}{P_{5}}\right).$$

(15) $$T_{5s}=T_{4}-T_{4}\eta_{hpt}\left(1-{\left(\frac{1}{PR_{hpt}}\right)}^{\frac{\left(\;\gamma_{g}-1\right)}{\gamma_{g}}}\right).\;\;\;$$

(16) $$T_{5}=T_{4}-T_{4}\eta_{hpt}\left(1-\left(\frac{T_{5s}}{T_{4}}\right)\right).$$

(17) $${\dot{W}}_{hpt}=\;{\dot{m}}_{2}\left(h(T_{4}\right)-h(T_{5})).$$

For LPT,

(18) $$\Psi \left(T_{6s}\right)=\Psi \left(T_{5}\right)+\ln\left(\frac{P_{5}}{P_{6}}\right).$$

(19) $$T_{6s}=T_{5}-T_{5}\eta_{lpt}\left(1-{\left(\frac{1}{PR_{hpt}}\right)}^{\frac{\left(\;\gamma_{g}-1\right)}{\gamma_{g}}}\right).\;\;\;$$

(20) $$T_{6}=T_{5}-T_{5}\eta_{lpt}\left(1-\left(\frac{T_{6s}}{T_{5}}\right)\right).$$

(21) $${\dot{W}}_{lpt}=\;{\dot{m}}_{2}\left(h(T_{5}\right)-h(T_{6})).$$

For the free power turbine, the same steps are repeated as follows:

(22) $$\Psi \left(T_{7s}\right)=\Psi \left(T_{6}\right)+\ln\left(\frac{P_{6}}{P_{7}}\right).$$

(23) $$T_{7s}=T_{6}-T_{6}\eta_{fpt}\left(1-{\left(\frac{1}{PR_{fpt}}\right)}^{\frac{\left(\;\gamma_{g}-1\right)}{\gamma_{g}}}\right).\;\;\;$$

(24) $$T_{7}=T_{6}-T_{6}\eta_{hpt}\left(1-\left(\frac{T_{7s}}{T_{6}}\right)\right).$$

(25) $${\dot{W}}_{fpt}=\;{\dot{m}}_{2}\left(h(T_{6}\right)-h(T_{7})).$$

For exhaust duct,

(26) $$P_{8}=P_{7}\times \left(1-\;\Delta PR_{exhaust}\right).$$

These design point calculations results were compared with results from a previous research conducted by Zhu and Saravanamuttoo [30] and data from the gas turbine product catalog. As far as credibility goes, the product catalog data is considered more reliable. Hence, both simulations results (i.e., from Zhu and Saravanamuttoo [30] and from the GasTurb 12 in the present study) were validated against the product catalogue data. As presented in Table 4, the percent error in estimated power output and exhaust temperature from the Zhu and Saravanamuttoo study showed significantly high deviation from the catalog data. On the contrary, the percent errors calculated for the results from GasTurb 12 in the present study showed only a very minute deviation from the catalogue data. Therefore, it was concluded that design point results generated through GasTurb 12 simulation are considered more reliable because they are meeting the actual design norms by showing less deviation. 

Once the cycle design point calculations were accurately estimated in GasTurb 12, suitable compressor and turbine performance maps were then selected. Following that, a parametric study on the effect of the ambient inlet air temperature (*T_amb_*) was performed in the range 265–315 K with an interval of 5 K. The temperature range was segregated into three scenarios: (i) Inlet air cooled temperature (*T_amb_ =* 265 K), (ii) near design point temperature (*T_amb_ =* 290 K), and (iii) hot day temperature (*T_amb_ =* 315 K). After setting these temperature ranges as an input to the simulation program, the off-design performance simulations were tracked.

### 2.3. Off-Design Simulations

During off-design simulations, the GasTurb 12 performs two major tasks: (i) Adaptation of the design point of the target engine with already known compressor and turbine maps using scaling method; and (ii) component matching by guaranteeing the compatibility of mass flow and work using Newton-Raphson iterative algorithm [29,31]. For compressor map adaptations, a suitable characteristics map with design point data was selected and then it was digitized in a form of a lookup table and finally exit calculations were performed. Auxiliary coordinate, *β*, was incorporated in the compressor map digitization process to avoid discrepancy as suggested by Kurzke [32]. After the inclusion of *β* coordinates, compressor characteristics were embodied as the following functions,

(27) $${\left(PR_{c}\right)}_{map}=f\left(\%N_{c},\;\beta_{c}\right),$$

(28) $${\left(\frac{\dot{m_{c}}\sqrt{\theta }}{\delta }\right)}_{map}\;=f\left(\%N_{c},\;\beta_{c}\right),$$

(29) $${\left(\eta_{c,isen}\right)}_{map}=f\left(\%N_{c},\;\beta_{c}\right),$$

where,

(30) $$\theta =\frac{T_{o}}{288.15\;K},$$

(31) $$\delta =\frac{P_{o}}{101.325\;KPa}.$$

Neverthless, due to unavailability of compressor maps in the literature, scaling was performed to make the design point of the desired engine to be in accordance with the design of the original compressor. GasTurb 12 utilizes the same scaling factors introduced by Sellers and Deniel [33], as given in Equations (32)–(34).

(32) $$PR_{c}=\frac{{\left(PR\right)}_{d}-1}{{\left(PR\right)}_{map,\;\;d}-1}\;\left[{\left(PR\right)}_{map}-1\right]+1.$$

(33) $$\frac{\dot{m_{c}}\sqrt{\theta }}{\delta }=\;\frac{{\left(\frac{\dot{m_{c}}\sqrt{\theta }}{\delta }\right)}_{d}}{{\left(\frac{\dot{m_{c}}\sqrt{\theta }}{\delta }\right)}_{map,d}}\times \;\dot{(m_{c}}\sqrt{\theta }/\delta \;{)}_{map}.$$

(34) $$\eta_{c,isen}=\;\frac{\eta_{d}}{\eta_{map,\;\;d}}\times \;\eta_{map}.$$

**Figure 2:**
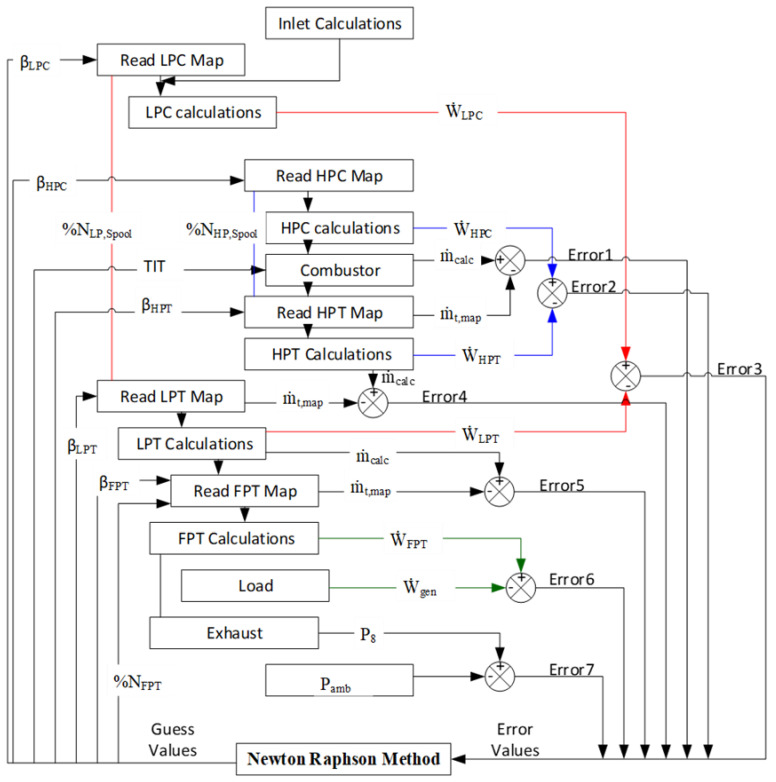
Newton-Raphson algorithm for component matching in GasTurb 12.

Component matching of all the components of a gas turbine is indispensable because it helps in estimating an optimized overall performance of the system at a specified ambient condition. In GasTurb 12, the components are matched by ensuring the compatibility of flow and work between the interconnected individual components. Hence, to develop a steady-state off-design operating line, the Newton-Raphson iterative algorithm was employed due to its inherent efficiency in numerical solution and ease of applicability to non-linear systems [29]. In the present study, seven iteration variables (*β_LPC_, β_HPC_, TIT, β_HPT_, β_LPT_, β_FPT_, %N_FPT_*) and seven errors were used to carry out the off-design calculation. The schematic diagram for component matching using Newton-Raphson method is shown in Figure 2. Mass flow and work compatibility equations are given in Equations (35)–(41).

(35) $${\dot{m}}_{2}-{\dot{m}}_{overboard}-{\dot{m}}_{cooling}=\;{\dot{m}}_{3}.$$

The combustor section,

(36) $${\dot{m}}_{3}+{\dot{m}}_{f}=\;{\dot{m}}_{4}.$$

The HP turbine section,

(37) $${\dot{m}}_{4}+{\dot{m}}_{NGV,hpt}+{\dot{m}}_{Rotor,htp}=\;{\dot{m}}_{5}.$$

The LP turbine section,

(38) $${\dot{m}}_{5}+{\dot{m}}_{NGV,lpt}+{\dot{m}}_{Rotor,ltp}=\;{\dot{m}}_{6}.$$

Apart from this, work compatibility between the components were ensured as follows:

For HP spool,

(39) $$W_{HPC}=W_{HPT}.$$

The LP spool,

(40) $$W_{LPC}=W_{LPT}.$$

The power turbine spool,

(41) $$W_{PT}=W_{Load}.$$

### 2.4. Variable Inlet Guide Vane Drift Simulations

**Figure 3:**
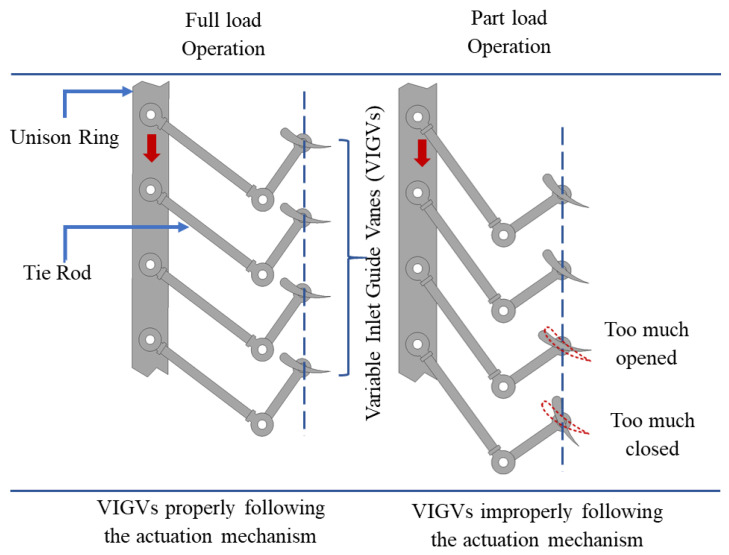
Variable inlet guide vane (VIGV) actuation mechanism and VIGV drift.

In general, faults associated with the variable geometry mechanism of the compressor are hard to detect during real-time system operation. As an alternative, some faults were implanted deliberately in order to observe the effects of these unforeseen faults on the severity of overall gas turbine’s performance deterioration. To investigate the effect of VIGV drift, a VIGV schedule suggested by Muir et al. [26] was considered as the benchmark and standard schedule for the present study. Drift in the variable geometry mechanism occurs typically when one or more of the guide vanes are not moving according to the schedule provided by the gas turbine control system, as shown in Figure 3. Occasionally, failure in some bolts or wearing of the links in the VIGV mechanism can also lead to a drift. That is the reason why the guide vanes that do not follow the control schedule are termed as mis-scheduled or drifted outside the normal schedule.

**Figure 4:**
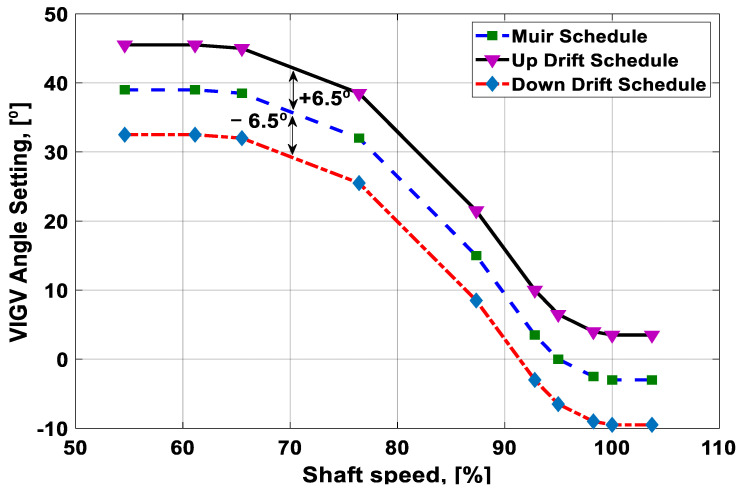
VIGV schedules utilized in the simulation.

In GasTurb 12, the variable guide vane drift were simulated by incorporating the VIGV schedule as an input command during the off-design simulation phase. These simulations were carried out for three different VIGV schedules: (i) down-drift schedule, (ii) normal schedule, and (iii) up-drift schedule. The schedules were established by featuring a ± 6.5° angle offset margin from the optimum schedule of Muir et al. [26]. The offset margin of *θ_VIGV_* = −6.5° was termed as the down-drift schedule, while *θ_VIGV_ =* +6.5° was named as up-drift from the Muir’s schedule. The reason for developing these two offset margins in the VIGV schedule was due to the fact that during real-time operation the gas turbine may approach these two limits: the maximum limit (+6.5°) and the minimum limit (−6.5°). The three variable guide vane schedules as a function of percent spool speed are shown in Figure 4.

Design point calculations in GasTurb 12 assumes a fixed geometry (*θ_VIGV_ =* 0°) compressor. To establish the off-design steady-state simulations, a VIGV schedule needs to be selected according to the configuration of the engine. The VIGV feature in GasTurb 12 is activated by assuming some suitable values for the correction factors, $a_{VIGV}$, $b_{VIGV}$, and $c_{VIGV}$, as given in Equations (41)–(44). Every 1° change in VIGV angle results in a 1% variation in the mass flow and pressure ratio. So an appropriate value of ∂VIGV (°) is always needed [28].

(42) $$a_{VIGV}=\;\frac{\partial \dot{m}\;\left(\%\right)}{\partial VIGV\;\left({}^{\circ}\right)}.$$

(43) $$b_{VIGV}=\;\frac{\partial \left(PR-1\right)\;\left(\%\right)}{\partial VIGV\;\left({}^{\circ}\right)}.$$

(44) $$c_{VIGV}=\;\frac{\partial \eta \;\left(\%\right)}{\partial VIGV\;\left({}^{\circ}\right)}.$$

The correction factor required for efficiency in GasTurb 12 is done by changing the $c_{VIGV}$ factor in the equation given below (Equation (45)). It became evident from a number of simulations that efficiency decreases by fraction of 0.01 for every ±10° modulation in ∂VIGV (°) setting, whereas a ±15° variation in the angle reduces efficiency by 0.0225 [28]. Hence, the assumed values $a_{VIGV}$ *=* $b_{VIGV}$ = 1 and $c_{VIGV}$ = 0.01 are kept constant as an input wherever more information about the compressor is not available, and this assumption also avoids excessive efficiency loss [28,29].

(45) $$\eta =\eta_{map}\times \left(1-VIGV^{2}\times \frac{c_{VIGV}}{100}\right).$$

In the present study, the variable guide vanes were considered for low pressure compressor as per the available specifications for LM1600 engine. The effect of performance deterioration was evident from compressor characteristics maps, as discussed in the following sections.

### 2.5. Effect of Inlet Air Temperature 

The effect of inlet air cooling was also accounted in the parametric study by designating a temperature of *T =* 265 K as the temperature after integrating the gas turbine system with inlet air cooling mechanism. However, there was no such physical or thermodynamic connection of IAC system with the studied gas turbine, rather IAC was visualized by setting a temperature of 265 K as the inlet cooled temperature in the parametric study. The details of the study are mentioned in the following sections.

### 2.6. Simulating the Effect of Fouling 

Other phenomena that may lead to performance deterioration in a gas turbine include fouling, erosion, corrosion, and foreign object damage (FOD) in the compressor and turbine sections. In this study, fouling in the compressor was taken into consideration. Compressor fouling takes place when some contaminated particles adhere to the surface of the blades and the annulus flow passage. The aerodynamic behavior may change due to the decreased flow passage owing to the particulate deposits. Compressor fouling can lead to (i) mass flow reduction, (ii) loss of pressure ratio and efficiency, (iii) increased heat rate, (iv) increased specific fuel consumption, and (v) reduced surge margin.

Compressor fouling was simulated in the GasTurb 12 using the *Modifier* option available in the software. Physical faults in the gas turbine were quantified by the change in the various independent parameters (i.e., isentropic efficiency, flow capacity, NGV area, and combustion efficiency etc.) that describe component performance. This change in the independent parameters then leads to deviation in the dependent parameters such as temperature, pressure, power output, and fuel flow. In the present study, change in the compressor isentropic efficiency *(∆η_c_*) and flow capacity *(∆*Γ_c_) (independent parameters) were considered to quantify the fouling phenomena in the compressor. Escher et al. [34] developed some correlations between the physical faults and the independent parameters. According to these correlations, for every 1% decrease in the compressor isentropic efficiency, the mass flow decreases by 3%. It is worth mentioning that the maximum interval limits for efficiency variation are 0% and −2.5%, while for compressor flow capacity it is 0% and −7.5%, maintaining a ratio of 1:3. To simulate the deterioration model, component characteristics maps need to be updated to accommodate for the deterioration. However, the maps already stored in the simulation program are for a clean engine. Thus, for a deteriorated engine the scaling factors of the compressor map need to be updated according to the variation of the independent parameters. The scaling factors for mass flow, isentropic efficiency, and pressure ratio as suggested by Qingcai et al. [16] are given below.

(46) $${\left(SF_{\Gamma }\right)}_{c}=1+\frac{{\Delta \Gamma }_{c}}{100}.$$

(47) $${\left(SF_{\eta }\right)}_{c}=1+\frac{\Delta \eta_{c}}{100}.$$

(48) $${\left(SF_{PR}\right)}_{c}=1+\frac{\Delta PR_{c}}{100}.$$

### 2.7. Effect of Fouling and VIGV Drift on Physical Parameters

Incipient faults in a gas turbine are generally traced using carefully selected indicators, which are based on the variations in the values of the dependent parameters. Dependent parameters are those that are measurable with the help of sensors at specific stations in the gas turbine. Pressure, temperature, fuel flow, and power output are considered dependent parameters. In case of any physical faults such as fouling, erosion and corrosion, or any malfunction (e.g., VIGV drift), these physical parameters are altered. A detailed comparison of all the physical parameters at every stage in the gas turbine is shown in Table 5. Fouling displayed a significant effect and hence it was quantified at two different severity levels: 0% and 100%. In addition, fouling was evaluated at three different schedules. With the increase in the fouling severity level, the mass flow and pressure showed a significant decrease at every station, while the temperature increased. The percent variation of pressure between the clean condition and fully deteriorated condition at the down-drift schedule was comparatively lower than the other two schedules. Similarly, the percent deviations for temperature at Muir’s schedule and the up-drift schedule were comparatively higher than at the down-drift schedule. It was inferred from this comparison that the Muir’s and the up-drift schedules were more vulnerable to fouling as compared to the down-drift schedule. The comparison in the table suggests that in the case of simultaneous fouling and VIGV drift, the probability of failure would be more at the VIGV up-drift and Muir’s schedule. Hence, the VIGV down drift was proved to be a good schedule in order to avoid failure due to fouling and VIGV drift. Moreover, this analysis of physical parameters at each station can be used for effective fault detection and diagnostics during the combined failure mode. 

## 3. Results and Discussion

### 3.1. Effect of the VIGV Drift on the Component Performance Maps

**Figure 5:**
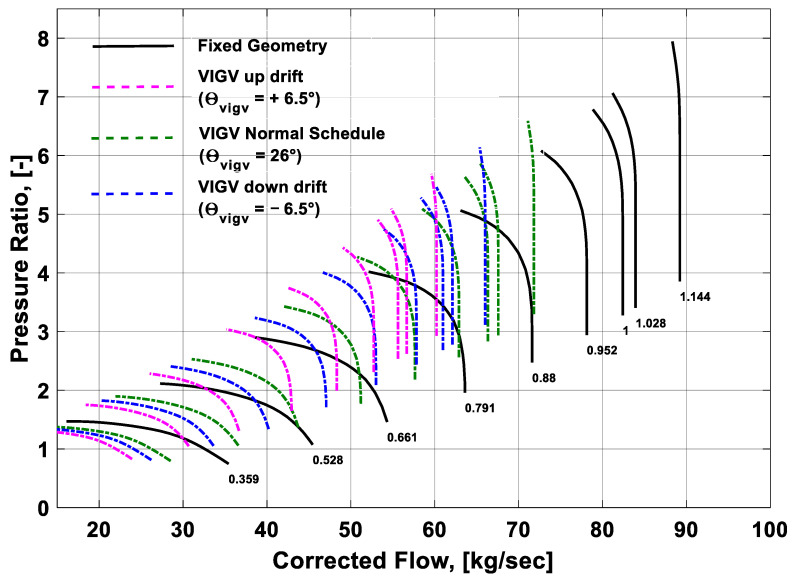
Effect of VIGV drift on pressure ratio.

The overall performance of a gas turbine is generally represented in terms of performance maps of its constituent components. The scope of this study was limited to the geometry of the LP compressor. Any small change in the geometry of the LP compressor initiates a new compressor and hence newer performance characteristics are obtained. A change in the variable geometry angles of the compressor vanes also depicts a new engine having varied performance characteristics than that of the older fixed geometry performance characteristics. In the present study, the performance maps for fixed geometry and three VIGV schedules of the LM1600 industrial gas turbine were generated from the simulation data. The performance map shown in Figure 5 represents the performance characteristics of the LP compressor at fixed geometry and VIGV drift phenomenon. Pressure ratio is a function of shaft speed and corrected mass flow. A random VIGV angle (i.e., $\theta_{VIGV}=26{}^{\circ}$) was selected from the Muir’s schedule. In order to quantify the maximum (up-drift) and minimum (down-drift) limits of the VIGV drift, an offset margin of ±6.5° was incorporated. The black lines represented the performance characteristics at the fixed geometry. This study revealed that incorporating the VIGV schedule suggested by Muir shifted the speed lines to the left side and slightly elevated from the speed lines at the Muir schedule, which showed that surge margin was enlarged. A rise in the surge margin was also noticed on the down-drift schedule at the midway speed rages of 79–88%. On the up-drift schedule the surge margin showed an improvement, but it was more prominent during the lower speed regimes on the bottom left corner. The other thing worth mentioning was that for every VIGV angle manipulation, the pressure ratio and corrected mass flow were also varied. Hence, both of the VIGV schedules were influencing the pressure ratio and corrected mass flow when the surge life shifts upwards. Accordingly, the pressure ratio increased, whereas the corrected mass flow of the compressor decreased. It is evident that both the VIGV drift schedules helped to manage the surge margin.

**Figure 6:**
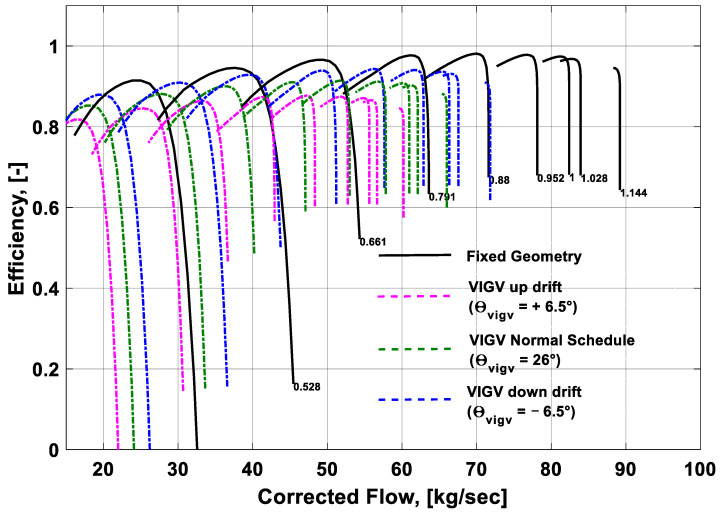
Effect of VIGV drift on efficiency.

Similarly, the performance of the compressor can also be visualized by another compressor map showing isentropic efficiency and corrected mass flow as a function of speed and auxiliary coordinate *β*. The performance map shown in Figure 6 depicts that at fixed geometry the isentropic efficiency was slightly more than the rest of the schedules. Usually, the VIGV schedule is adopted to manipulate the flow of the incoming air during part load scenario and shaft speed is desired to be decreased. That is why at Muir’s schedule the isentropic efficiency of the LP compressor is showing a substantial decrease than that of the fixed geometry efficiency. The VIGV drift schedules were defined by keeping an offset of +6.5° for up-drift and −6.5° for down-drift in reference to the Muir’s schedule. The angle that was considered from the Muir’s schedule was $\theta_{vigv}=26{}^{\circ}$ and it was chosen randomly at a certain speed. The interesting thing to note was that the VIGV down-drift schedule maintained a slightly higher isentropic efficiency than the Muir’s schedule, whereas the VIGV up-drift schedule showed a lowest isentropic efficiency among all the VIGV schedules. As the speed lines shifted to the left side, the corrected mass flow happened to decrease in all the schedules. After the analysis of the compressor maps, it was concluded that the VIGV down-drift schedule can provide good stability in terms of an improved surge margin. 

### 3.2. Effect of Fouling on Component Performance Maps

**Figure 7:**
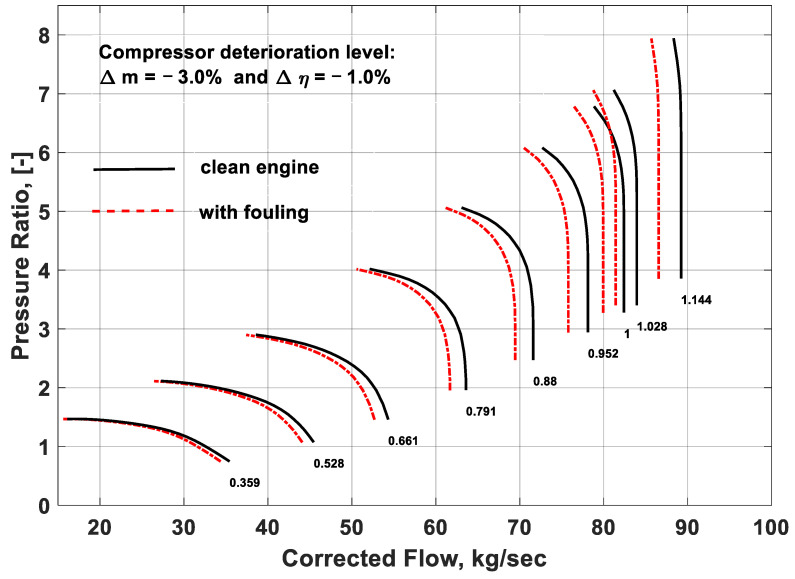
Effect of fouling on pressure ratio.

**Figure 8:**
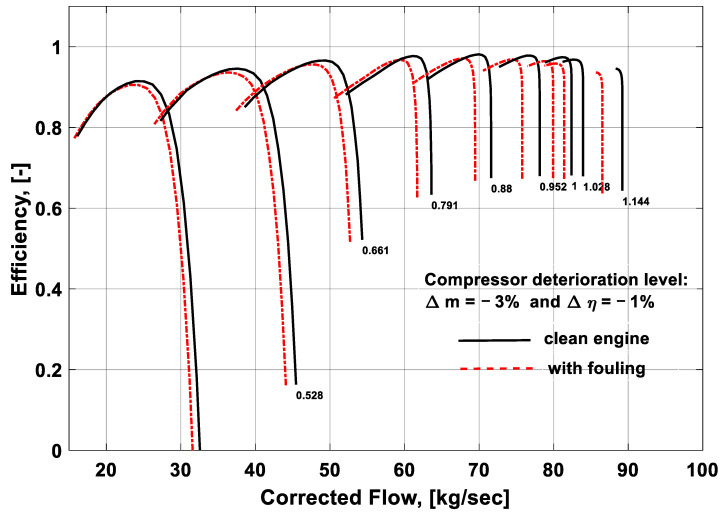
Effect of fouling on efficiency.

During compressor fouling, the aerodynamic shape and geometry of the compressor are changed, which may lead to a change in the performance characteristics feature of the component. Hence, the component characteristics curves are shifted from clean to deteriorated condition due to the change in the operating point owing to the compressor fouling, as shown in Figure 7 and Figure 8. The black lines in the performance map indicate the clean compressor conditions, while the red lines show the deteriorated condition due to fouling. The fouling severity was simulated by quantifying the deterioration rate of the independent parameters such as flow capacity and isentropic efficiency. From Figure 7, it can seen that, due to the compressor fouling, the pressure ratio and corrected mass flow deviated from the clean compressor conditions. The reason lies on the fact that a fouled compressor behaves like a new engine and the operating range is reduced that shifted the engine operating point toward the lower mass flow rates at any certain speed. In this way, it also changes the operating line of the compressor. Mohammadi and Muntazari [17] reported that a change in pressure ratio is a function of fouling severity and operating point as ∆*PR* = *f* (∆Γ,*K*), where *K* is the slope of the *β*-line that represents change in operating point due to updated component matching. This change in the operating point from clean to fouled conditions defines a new engine characteristic that shows substantial deviations from the original behavior. It is evident from the performance map that the pressure ratio and corrected mass flow values are experiencing deterioration due to fouling. These deteriorated values can eventually degrade the engine performance. Apart from this, surging can also happen that may lead to LP compressor failure and engine’s shut down. These kind of performance maps are very helpful in gas turbine fault detection and diagnostics. 

Compressor characteristics curves can also be represented in the form of efficiency and corrected mass flow as a function of shaft speed and beta coordinate, *β*. Just like the previous performance map, here also the fouling plays its role in changing the geometry of the compressor. This change in geometry leads to a change in the operating point of the engine and in turn the operating line on the LPC map. As shown in Figure 8, fouling was quantified by changing the values of the independent parameters (i.e., Δ*m* = −3% and Δ*η* = −1%). The simulated results showed a deviation from the clean condition, depicted by the black curves in Figure 8, as represented by the red curves. It was inferred from the compressor performance maps that due to fouling the isentropic efficiency and corrected mass flow decreased that can deteriorate the overall performance of the engine. Moreover, due to fouling the annulus passage of the air inside the LPC reduces and it may trigger surging that can eventually lead to compressor failure and plant shutdown. This study also revealed that the effect of fouling on the LPC performance parameters, isentropic efficiency, and corrected mass flow was more rigorous at higher shaft speeds compared to the lower shaft speeds. Moreover, at higher speeds, the constant speed lines became steeper on the compressor map due to the increased roughness attributable to fouling. Thus, the range of the mass flow covering the characteristics curves and pressure ratio was reduced. Hence, it was concluded that at high shaft speed, the probability of surging in LPC increased due to fouling. That is the reason why there is a need to develop simulation models for effective prediction of fouling in industrial gas turbines for reliable and safe operations.

### 3.3. Combined Effect Analysis

**Figure 9:**
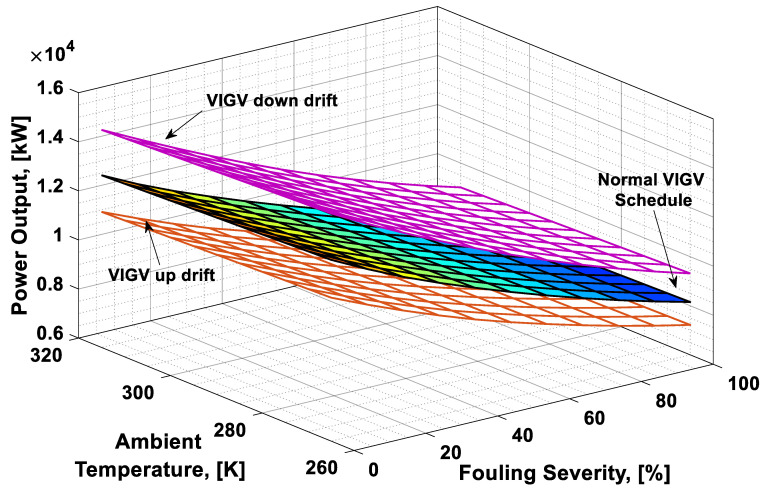
Combined effect of performance deterioration phenomena on power output.

**Figure 10:**
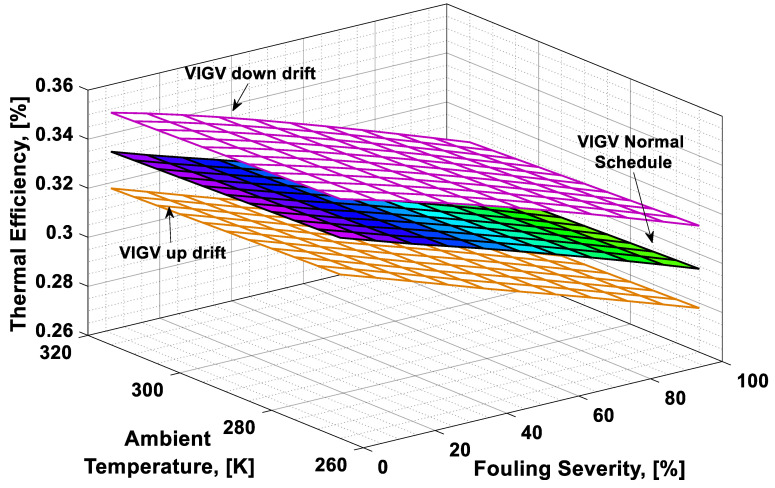
Combined effect of performance deterioration phenomena on thermal efficiency.

**Figure 11:**
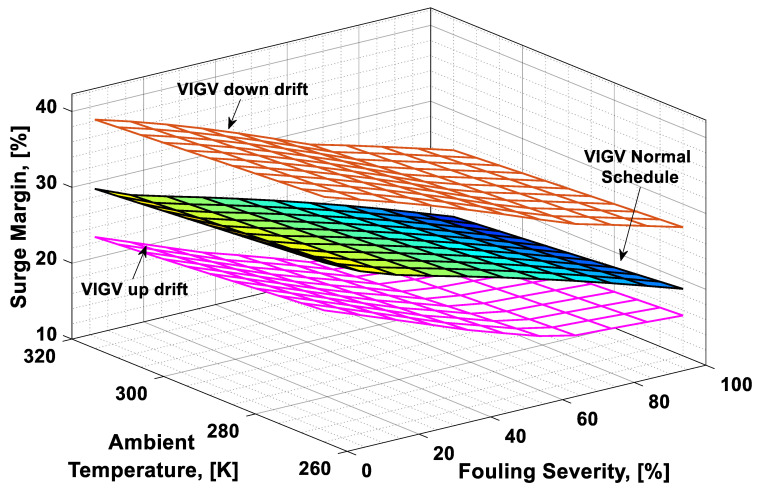
Combined effect of performance deterioration phenomena on surge margin.

**Figure 12:**
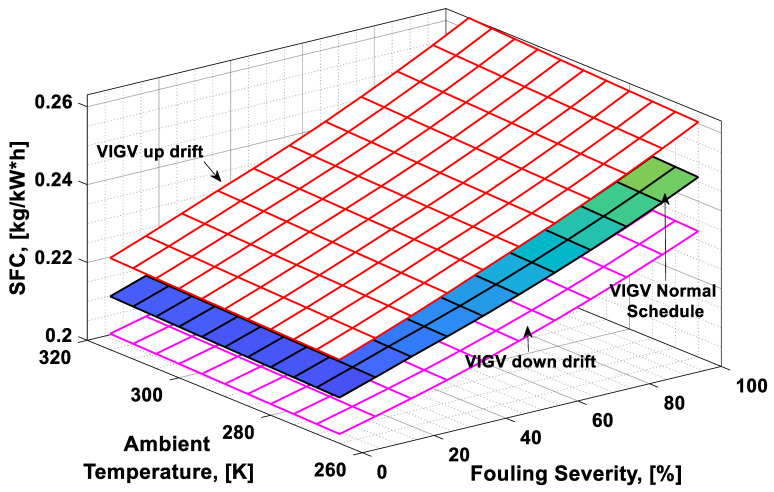
Combined effect of performance deterioration phenomena on specific fuel consumption (SFC).

**Figure 13:**
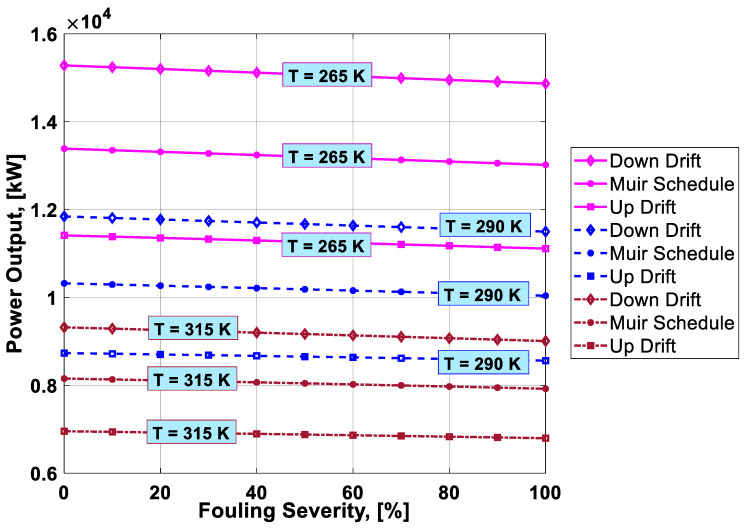
Combined effect of inlet air cooling (IAC), fouling, and VIGV drift on power output.

**Figure 14:**
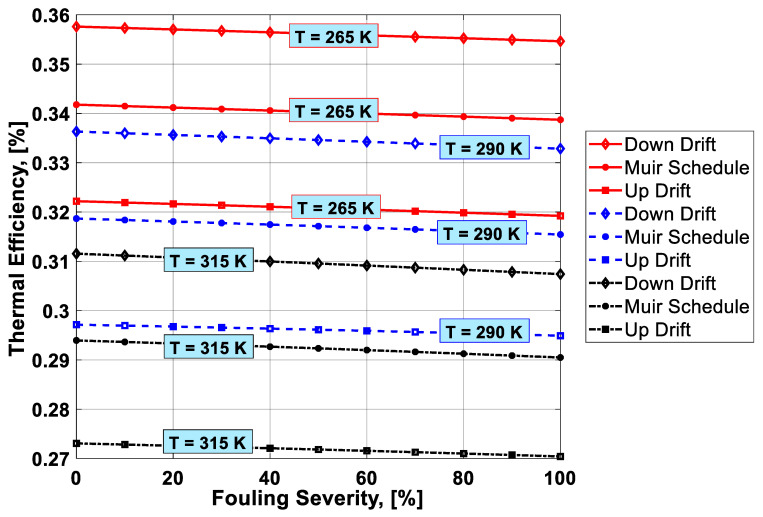
Combined effect of IAC, fouling, and VIGV drift on thermal efficiency.

**Figure 15:**
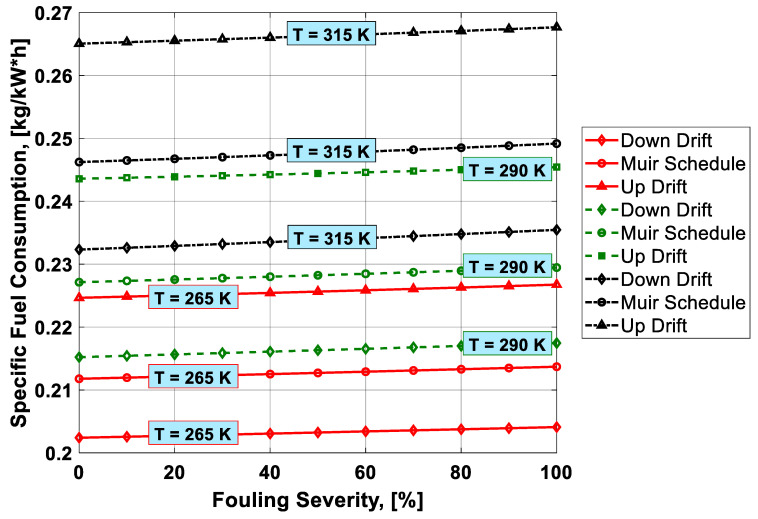
Combined effect of IAC, fouling, and VIGV drift on specific fuel consumption (SFC).

**Figure 16:**
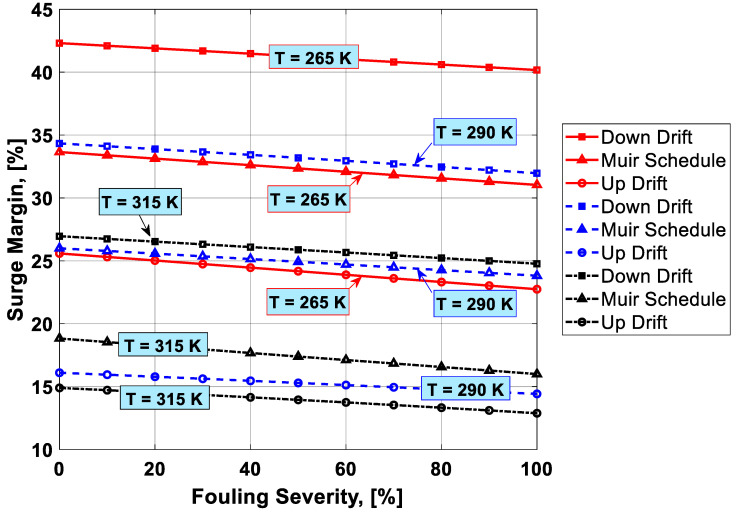
Combined effect of IAC, fouling, and VIGV drift on surge margin.

To visualize the combined effect of the three performance deterioration parameters, two types of plots (surface and 2D plots) were developed from the simulation data. The surface plots (Figure 9, Figure 10, Figure 11 and Figure 12) were used to observe the combined effect of VIGV drift, fouling severity, and ambient temperature. Although, surface plots were able to represent a clear picture of the effects of VIGV drift and fouling on performance parameters, the effect of ambient temperature on performance were effectively envisaged from the 2D plots (Figure 13, Figure 14, Figure 15 and Figure 16). 

It was observed that with the increase in the fouling severity level from 0% to 100%, the power output, thermal efficiency, and surge margin reduced linearly as shown in Figure 9, Figure 10 and Figure 11. With the increase in the fouling severity level, the power output and thermal efficiency of the overall system exhibited continuous deterioration. The surge margin in the LPC also decreased with the increase of fouling severity, as shown in Figure 11. The drift phenomenon was depicted by describing a deviation of VIGV schedule from the normal schedule. These deviations were termed as (i) up-drift schedule and (ii) down-drift schedule. During the up-drift schedule the power output happened to be deteriorated by 15.38%, while at the VIGV down-drift schedule the power output seemed to be improved by 14.75% during clean compressor conditions. Whereas during 100% fouling severity level, 14.72% of power was degraded at up-drift, while at down-drift 14.53% power was augmented, as shown in Figure 9. However, with the increase of fouling severity the power output deteriorated at its respective schedule. Similarly, thermal efficiency during up-drift was showing a loss of 6.74% from that of normal schedule efficiency, while during down-drift schedule, a 5.55% rise in thermal efficiency was noticed during clean condition of the compressor, whereas these deviation rates increased during 100% fouling severity level. Hence it became clear that thermal efficiency also showed deterioration linearly, with the increase of fouling severity level, as shown in Figure 10. Apart from this surge margin in low pressure compressor decreased with passage of deterioration. During up-drift schedule the surge margin happened to be diminished by 38.05%, whereas during down-drift phase it appeared to be improved by 32.08%, which can avert LPC from surging substantially. On the contrary, specific fuel consumption was noticed as increasing with fouling severity level, as depicted in Figure 12. However, in the down-drift case, it seemed to be improved by 5.23%, which is economically viable for power generation companies.

As far as the effect of ambient temperature on performance parameters was concerned, a temperature range was allocated in the simulation program by conducting a parametric study for inlet air ambient temperature (*T_amb_*). The upper limit for the ambient temperature was set as (*T =* 315 K) representing a hot day temperature in any tropical climate region. The temperature (*T =* 290 K) was defined as “near design point”. This is representing the normal temperature that an engine may face during its operation. It can be clearly observed from Figure 9, Figure 10 and Figure 11 that, although the performance deteriorated with increasing level of fouling severity (SF%), during a hot day temperature (*T =* 315 K) the deterioration rate was much larger than that of near design point. Thus, it became evident, during a hot day in a tropical region the performance deteriorated due to increased inlet ambient temperature. Apart from this, surge margin also decreased at high ambient temperatures. Hence it was concluded that high ambient temperature led to performance deterioration. It also increased failure rate of the compressor due to a surge that may create high maintenance cost. On the other hand, specific fuel consumption increased with increasing fouling severity level. This SFC increased more drastically at increased ambient temperature (*T =* 315 K). During down-drift, an even more rigorous decrease in SFC was observed as shown in Figure 12.

Inlet air cooling (IAC) is a commercially adopted technique to improve the performance of a gas turbine caused by high ambient temperatures. In the present study, a temperature setting of *T =* 265 K was allocated as inlet cooled temperature. Although, there was no IAC physically installed as part of the gas turbine, for simulation purposes a temperature as low as *T =* 265 K was idealized as the temperature achieved after integrating a cooling mechanism at the inlet of the simulated gas turbine. It is clear from the Figure 13, Figure 14, Figure 15 and Figure 16 that power output, thermal efficiency, and surge margin were improved by 29.67%, 7.38%, and 32.84%, respectively, at temperature *T =* 265 K after the integration of the inlet air cooling mechanism. However, previously these performance parameters showed substantial deterioration (i.e., 21.09%, 7.92%, and 30.32%, respectively) due to high ambient temperature and fouling, as indicated in Table 6. Apart from this, specific fuel consumption rate decreased by 6.88% due to integration of an IAC technique that is economically good. Figure 15 clearly portrayed the improvement in SFC – the SFC lines shifted from top to bottom at a temperature setting of *T =* 265 K. It was concluded from the above discussion that the down-drift schedule scenario improved the performance that was threatened by increased ambient temperature and fouling. In addition, in cases where an IAC mechanism should be integrated with the gas turbine operating in hot climates, the performance improved in a better way, as shown in Figure 13, Figure 14, Figure 15 and Figure 16.

Combined effect analysis revealed that, although the performance deteriorated due to fouling, each VIGV drift schedule and temperature showed a different performance. For instance, at high ambinet temperature (*T =* 315 K), the difference in performance at each VIGV schedule was comparatively smaller than that at IAC temperature (*T =* 265 K), as shown in Figure 13 and Figure 14. Similarly, for SFC, the VIGV down-drift schedule was showing more deviation from the Muir schedule, while up-drift indicated more deviation at both temperatures (*T =* 265 K) and (*T =* 315 K), but there was uniform deviation in the SFC values at each VIGV schedule at near design point temperature, as shown in Figure 15. However, for surge margin, VIGV down-drift and up-drift schedules had very little deviation from Muir at (*T =* 265 K) as the temperature was increased, in that, at *T =* 265 K and *T =* 315 K, the deviations of each schedules showed a significant difference from that of normal schedule as shown in Figure 16. Thus, it was observed that at low temperature the deviation was normal but as the temperature goes up the deviations in the performance values at their respective schedule would rise. 

## 4. Conclusions

In the present study, modeling and steady-state off-design simulation were performed for a three-shaft industrial gas turbine (aeroderivative gas turbine, GE LM1600) to investigate the combined effect of VIGV drift, fouling, and inlet air cooling on the overall performance of the engine. The simulation model was developed with the help of a commercially available software GasTurb 12. VIGV drift was simulated by considering two different schedules deviating from the normal schedule that was adopted from Muir study. Apart from this, fouling was simulated by running the simulation model of 11 different fouling severity levels from 0% to 100%. These fouling severity levels were incorporated by two independent health parameters (i.e., change in compressor flow capacity and change in compressor isentropic efficiency). With increasing levels of fouling severity, the performance parameters showed a significant deterioration. However, combining the VIGV down-drift schedule resulted in increased power output, thermal efficiency, and surge margin by 14.53%, 5.55%, and 32.08%, respectively. Similarly, SFC was also decreased by 5.23% at VIGV down-drift schedule. In addition, a parametric study was conducted in order to see the effect of increased inlet air temperature on the performance. During parametric study, three temperature settings were defined to indicate different ambient condition. It became evident that, with increased temperature (*T* = 315 K) and increased fouling severity level, the overall performance deteriorated rigorously. Consequently, the down-drift schedule helped in managing the performance to an improved level. A temperature of *T* = 265 K was considered as inlet air cooled temperature, obtained right after the integration of gas turbine inlet with an inlet air cooling mechanism. The integration of the inlet air cooling technique helped to improve the power output, thermal efficiency, surge margin, and SFC by 29.67%, 7.38%, 32.84%, and 6.88%, respectively. The simulation results revealed that the VIGV down-drift can compensate the effect of performance deterioration and compressor surge due to fouling and helps in improving the performance. 

The results from the developed model can serve as a basis for acquiring insights related to gas turbine fault detection and diagnostics (FDDs). Moreover, it also help in troubleshooting the root cause of the performance degradation and compressor surge in an engine faced with a VIGV drift and fouling simultaneously. 

## Figures and Tables

**Table 1:** Technical data for GE LM1600.

Parameter Name	Designated Value	Units
**Power Rating**	13,748	kW
**Heat Rate**	10,286	kJ/kWh
**Thermal Efficiency**	35%	-
**Overall Pressure Ratio**	20.2:1	-
**Exhaust Mass Flow**	47.3	kg/s
**Power Turbine Speed**	7900	RPM
**Exhaust Gas Temperature**	764.15	K

**Table 2:** Coefficients to calculate specific heats of air and combustion products [27].

Component	A	B	C
**O_2_**	936	13.1	−523
**N_2_**	1020	13.4	−179
**CO_2_**	1005	20	−1959
**Ar**	521	0	0

**Table 3:** Design point input data for LM1600.

Required Parameter Type	Parameter Name	Nomenclature	Value
**Ambient Conditions**	Ambient Temperature	*T*_amb_	288.15
	Ambient Pressure	*P*_amb_	101.325
	Relative Humidity	RH	60%
**Inlet Conditions**	Inlet Pressure Loss	∆P_1_	0.02
	Inlet Air Mass Flow	m_1_	46.75
**LP Compressor Input Data**	Pressure Ratio	PR_LPC_	4
	Polytropic Efficiency	*η*_poly_	0.91
	Bleed Fraction	bleed	0
	Shaft Speed	RPM	7000
**HP Compressor Input Data**	Pressure Ratio	PR_HPC_	5.05
	Polytropic Efficiency	*η*_poly_	0.8784
	Bleed Fraction	bleed	0.094
	Shaft Speed	RPM	12,000
**Burner Input Data**	Combustion Efficiency	*η*_comb_	0.99
	Pressure Loss	∆P_comb_	0.05
**Turbine Input Data**	Turbine Inlet Temperature	TIT	1419.92
	HP Turbine Polytropic Efficiency	*η*_poly_	0.8949
	LP Turbine Polytropic Efficiency	*η*_poly_	0.8937
	Free Power (FP) Turbine Polytropic Efficiency	*η*_poly_	0.8907
	FP Turbine Shaft Speed	RPM	7900
**Exhaust Duct Input Data**	Pressure Loss	∆P_8_	0.01
**Mechanical Efficiencies**	HP Spool	-	0.99
	LP Spool	-	1
	Generator Efficiency	-	1

**Table 4:** Validation of design point results obtained by optimization process in GasTurb 12.

Parameter	Units	Catalogue	Zhu and Saravanamuttoo [30]	% Error	GasTurb12 Simulation	% Error
**Power Output**	kW	13,748	12,312	10.45	13,683.5	0.47
**Thermal Efficiency**	%	35	40	14.28	35.41	1.17
**Pressure ratio**	-	20.2	22	8.91	20.2	0
**Exhaust Flow**	kg/s	47.3	43.78	7.44	47.3	0
**Exhaust Temperature**	K	764.15	736	3.68	766.1	0.26
**Heat rate**	kJ/(kW*h)	10,286	10,457	1.66	10,166	1.17

**Table 5:** Effect of fouling and VIGV drift on physical parameters.

			Down-Drift Schedule (−6.5°)		Muir’s Schedule		Up-Drift Schedule (+6.5°)	
Station No.		SF	0%	100%	0%	100%	0%	100%
	Physical Parameters							
1	*m*_1_ (kg/s)		41.622	40.867	38.807	38.177	34.992	34.611
	*P*_1_ (kPa)		339.582	335.242	313.373	310.320	277.620	276.939
	*T*_1_ (K)		431.914	433.618	428.088	430.228	421.259	424.218
2	*m*_2_ (kg/s)		40.623	39.886	37.875	37.261	34.152	33.780
	*P*_2_ (kPa)		1759.892	1727.923	1640.720	1614.071	1479.323	1463.128
	*T*_2_ (K)		729.585	730.355	727.123	728.077	723.023	724.192
3	*m*_3_ (kg/s)		38.197	37.503	35.616	35.037	32.119	31.768
	*P*_3_ (kPa)		1637.327	1607.453	1526.824	1501.873	1377.192	1361.942
	*T*_3_ (K)		1419.814	1419.840	1419.733	1419.765	1419.599	1419.638
4	*m*_4_ (kg/s)		39.862	39.138	37.168	36.564	33.519	33.152
	*P*_4_ (kPa)		620.414	611.024	576.081	568.962	515.036	512.530
	*T*_4_ (K)		1151.298	1152.075	1150.213	1151.200	1148.018	1149.511
5	*m*_5_ (kg/s)		41.194	40.446	38.410	37.786	34.638	34.260
	*P*_5_ (kPa)		361.795	355.389	338.800	333.463	308.095	304.844
	*T*_5_ (K)		1145.649	1146.428	1144.548	1145.537	1142.330	1143.823
6	*m*_6_ (kg/s)		41.694	40.936	38.876	38.244	35.058	34.675
	*P*_6_ (kPa)		351.033	344.834	328.787	323.626	299.109	295.968
	*T*_6_ (K)		1021.544	1020.885	1023.682	1022.865	1027.239	1026.238
7	*m*_7_ (kg/s)		42.110	41.345	39.264	38.626	35.408	35.021
	*P*_7_ (kPa)		106.326	106.160	105.747	105.616	105.013	104.937
	*T*_7_ (K)		784.952	786.991	796.540	798.299	814.311	815.18353

**Table 6:** Comparison of performance parameters deviations from design point conditions.

	Percent Deviation from the Design Point Conditions (T_o_ = 290 K, P_o_ = 101.325 kPa)			
	Inlet Air Cooling (265 K)		Hot Climate (315 K)	
	Clean Condition	Deteriorated Condition (100%)	Clean Condition	Deteriorated Condition (100%)
**Power Output (kW)**	29.70%↑	29.67%↑	20.99%↓	21.09%↓
**Thermal Efficiency (%)**	7.25%↑	7.38%↑	7.75%↓	7.92%↓
**Surge Margin (%)**	29.43%↑	32.84%↑	27.58%↓	30.32%↓
**SFC (kg/kWh)**	6.78%↓	6.88%↓	8.41% ↑	8.58%↑

## Nomenclature

**Nomenclature**	
*P*_*amb*_	Ambient pressure (Pa)
*T*_*amb*_	Ambient temperature (K)
${\dot{m}}_{a}$	Ambient mass flow rate (kg/s)
*c*_*pa*_	Specific heat for air (kJ/kg.K)
*c*_*pg*_	Specific heat for gaseous products (kJ/kg.K)
$\dot{W}$	Power consumed (kW)
*∆PR*	Pressure loss across intake duct
${\dot{m}}_{f}$	Fuel mass flowrate (kg/s)
*%N*	Percent spool speed
*h*	Enthalpy (kJ/kg)
${\dot{m}}_{\mathrm{exhaust}}$	Exhaust mass flow (kg/s)
W	Work
**Subscripts**	
*a*	Air
*amb*	Ambient
*s*	Isentropic value
*c, d*	Design point of target compressor
*map,d*	Design point of the compressor with performance map
*cc*	Combustion chamber
*isen*	Isentropic
*th*	Thermal
*a,1,2,3,4,5,6,7,8*	Station numbering
*Load*	Generator load
**Abbreviations**	
*PR*	Pressure ratio
*IAC*	Inlet air cooling
*VIGV*	Variable inlet guide vanes
*VSV*	Variable stator vanes
*SF*	Fouling severity
*LHV*	Lower heating value
*NGV*	Nozzle guide vane
*SFC*	Specific fuel consumption
VIGV	Variable guide vane
*HPC*	High pressure compressor
*LPC*	Low pressure compressor
*HPT*	High pressure turbine
*LPT*	Low pressure turbine
*PT*	Power turbine
*RVDT*	Rotary variable displacement transducer
**Greek letters**	
*β*	Beta values on compressor maps
*θ*	Temperature correction factor
*δ*	Pressure correction factor
*η*	Efficiency
*Γ*	Flow capacity
*∆*	Change
∂	Partial change
*Ψ*	Specific entropy
*γ*	Specific heat ratio
